# Draft Genome Sequence of Escherichia coli UMB1353, Isolated from the Female Urinary Tract

**DOI:** 10.1128/MRA.00416-20

**Published:** 2020-06-04

**Authors:** Tanea Crawford, Taylor Miller-Ensminger, Adelina Voukadinova, Alan J. Wolfe, Catherine Putonti

**Affiliations:** a Neuroscience Program, Loyola University Chicago, Chicago, Illinois, USA; b Bioinformatics Program, Loyola University Chicago, Chicago, Illinois, USA; c Department of Microbiology and Immunology, Stritch School of Medicine, Loyola University Chicago, Maywood, Illinois, USA; d Department of Biology, Loyola University Chicago, Chicago, Illinois, USA; e Department of Computer Science, Loyola University Chicago, Chicago, Illinois, USA; University of Maryland School of Medicine

## Abstract

Here, we present the draft genome sequence of Escherichia coli UMB1353, isolated from a patient with a urinary tract infection. The sequence of this antibiotic-resistant E. coli strain contains one intact P2-like phage.

## ANNOUNCEMENT

Escherichia coli is a Gram-negative, rod-shaped, facultative, anaerobic bacterium commonly found in the human gastrointestinal tract (1). While often a commensal species in this niche, it can cause meningitis, gastrointestinal symptoms and disorders, and urinary tract infections (UTIs) (2). UTI is one of the most common human infections (3, 4), and E. coli is the leading cause of UTIs (5). Despite many genome-sequencing projects of commensal and uropathogenic E. coli, the distinction between the two has yet to be determined (6). Here, we present the draft genome sequence of E. coli UMB1353, a strain isolated from a transurethral catheter urine sample obtained from a woman with a UTI.

Using the expanded quantitative urine culture (EQUC) method (7), E. coli UMB1353 was isolated as part of a prior institutional review board (IRB)-approved study (8). This urine specimen was collected from a woman seeking clinical care at Loyola University Medical Center’s Female Pelvic Medicine and Reconstructive Surgery Center (Maywood, IL, USA) from June 2014 to August 2015. The genus and species were identified using matrix-assisted laser desorption ionization–time of flight (MALDI-TOF) mass spectrometry before being stored at −80°C. E. coli UMB1353 was streaked onto a Columbia nalidixic acid (CNA) plate and incubated at 35°C with 5% CO_2_. A single flat colony was recovered and placed in LB broth and incubated at 37°C with shaking for 24 h. The DNA was extracted with the Qiagen DNeasy blood and tissue kit using the protocol for Gram-positive bacteria with the following modifications: we used 230 μl of lysis buffer (including 50 μl of lysozyme) in step 2 and incubated for 10 min at 56°C in step 5. Extracted DNA was quantified with a Qubit fluorometer. Library preparation and sequencing were done at the Microbial Genome Sequencing Center at the University of Pittsburg. First, the DNA was enzymatically fragmented using an Illumina tagmentation enzyme. Indices were attached using PCR, and then the library was sequenced on the Illumina NextSeq 550 platform. Sequencing produced a total of 2,302,396 pairs of 150-bp reads. Raw reads were trimmed using Sickle v1.33 (https://github.com/najoshi/sickle). The trimmed reads were assembled using SPAdes v3.13.0 with the “only-assembler” option for k values of 55, 77, 99, and 127 (9). Genome coverage was calculated using BBMap v38.47 (https://sourceforge.net/projects/bbmap/). Annotations were produced using PATRIC v3.6.3 (10), but the publicly available genome was annotated using the NCBI Prokaryotic Genome Annotation Pipeline (PGAP) v4.11 (11). Unless previously noted, default parameters were used for each software tool.

The assembled draft genome is 5,282,250 bp long with a GC content of 50.25%, genome coverage of 82×, and *N*_50_ score of 89,083 bp. The genome is composed of 195 contigs encoding 4,867 protein-coding genes per the PGAP annotation. PATRIC identified several transporter genes, drug target genes, and antibiotic resistance genes. Further investigation of the antibiotic resistance genes using ResFinder v3.2 (12) identified those for beta-lactams, macrolides, and tetracycline. PHASTER (13) identified one intact phage, similar to *Enterobacteria* phage P2. This was confirmed by querying the predicted prophage nucleotide sequence against the nonredundant nucleotide (nr/nt) database via MegaBLAST (GenBank accession no. LR595869.1; query coverage, 81%; percent identity, 97.62%). Analysis of this genome builds on previous research of uropathogenic E. coli and has clinical relevance by contributing to research for developing new UTI treatments.

### Data availability.

This whole-genome shotgun project has been deposited in GenBank under the accession no. JAAUWE000000000. The version described in this paper is the first version, JAAUWE010000000. The raw sequencing reads have been deposited in the SRA under the accession no. SRR11441035.
